# Sulforaphane Promotes Dendritic Cell Stimulatory Capacity Through Modulation of Regulatory Molecules, JAK/STAT3- and MicroRNA-Signaling

**DOI:** 10.3389/fimmu.2020.589818

**Published:** 2020-10-30

**Authors:** Yangyi Wang, Emilia Petrikova, Wolfgang Gross, Carsten Sticht, Norbert Gretz, Ingrid Herr, Svetlana Karakhanova

**Affiliations:** ^1^ Section Surgical Research, Molecular OncoSurgery Group, Department of General, Visceral and Transplantation Surgery, University of Heidelberg, Heidelberg, Germany; ^2^ Medical Research Center, Medical Faculty Mannheim, University of Heidelberg, Mannheim, Germany

**Keywords:** sulforaphane, dendritic cells, T cells, regulatory molecules, STAT3, miRNAs, pancreatic cancer

## Abstract

**Introduction:**

The broccoli isothiocyanate sulforaphane was shown to inhibit inflammation and tumor progression, also in pancreatic cancer, while its effect on tumor immunity is poorly understood. We investigated the immunoregulatory effect of sulforaphane on human dendritic cells alone and in presence of pancreatic tumor antigens, as well as underlying molecular mechanisms.

**Methods:**

Sulforaphane-treated human dendritic cells were matured *in vitro* with a cytokine cocktail, and the expression of regulatory molecules was examined by flow cytometry. The subsequent T-cell response was analyzed by T-cell proliferation assay and CD25 expression. To confirm the findings, dendritic cells pulsed with pancreatic cancer-derived tumor antigens were used. To identify the involved pathway- and microRNA-signaling in sulforaphane-treated dendritic cells, inhibitors of various signaling pathways, western blot analysis, microRNA array, and bioinformatic analysis were applied.

**Results:**

Sulforaphane modulated the expression of the costimulatory CD80, CD83 and the suppressive B7-H1 molecules on dendritic cells and thereby promoted activation of T cells. The effect was verified in presence of pancreatic tumor antigens. Phosphorylation of STAT3 in dendritic cells was diminished by sulforaphane, and the inhibition of JAK/STAT3 led to downregulation of B7-H1 expression. Among the identified top 100 significant microRNA candidates, the inhibition of miR-155-5p, important for the expression of costimulatory molecules, and the induction of miR-194-5p, targeting the B7-H1 gene, were induced by sulforaphane.

**Conclusion:**

Our findings demonstrate that sulforaphane promotes T-cell activation by dendritic cells through the modulation of regulatory molecules, JAK/STAT3- and microRNA-signaling in healthy conditions and in context of pancreatic cancer-derived antigens. They explore the immunoregulatory properties of sulforaphane and justify further research on nutritional strategies in the co-treatment of cancer.

## Introduction

Sulforaphane, a natural isothiocyanate present abundantly in cruciferous vegetables, such as broccoli, attracted considerable attention in oncology first because of epidemiological studies. They demonstrated that diets rich in sulforaphane-containing vegetables of the *Brassicaceae* family can lower the incidences of various cancer types, including pancreatic cancer (1).

Pancreatic ductal adenocarcinoma (PDA) is one of the most lethal malignancies worldwide with a poor survival rate and limited treatment options due to its pronounced therapy resistance and immunosuppressive nature (2–4). We demonstrated previously that purified sulforaphane is able to overcome therapy resistance in PDA as well as in prostate cancer *in vivo* and *in vitro* (5, 6), and a similar effect was confirmed in breast cancer (7, 8). Meanwhile, a phase II clinical trial with 20 patients suffering from recurrent prostate cancer suggested a suppressive effect of sulforaphane-rich broccoli sprout extract on the expression of the prostate-specific tumor progression marker PSA (9). Besides, Tahata et al. demonstrated its positive association with the expression of the tumor suppressor decorin in melanoma patients (10). Data of our recent prospective pilot study conducted with 40 patients with advanced non-resectable PDA point to a survival advantage after consuming sulforaphane-rich broccoli sprout powder (11).

Despite these encouraging results, immunoregulatory effects of sulforaphane remain largely unknown. Limited studies in various diseases and *in vitro* models reported both immunoactivating (12–14) and immunosuppressive properties (10, 15–18). This is of highest relevance because the ratio between immune activation and suppression plays a crucial role in cancer treatment. Although, sulforaphane-enriched supplements may support the therapeutic effect in cancer patients, its immunoregulatory potential is still under debate (19) and requires further elucidation.

A major part of immune activation is provided by dendritic cells (DCs), which serve as an immune response initiator and are involved in provoking both innate and adaptive immunity. In inflammatory conditions including tumors, antigen sampling is mainly mediated by DCs, especially by those that differentiate from monocytes recruited from the blood. DCs further proceed with activating T cells, aiming at tumor clearance, thus inducing an effective antitumor response (20).

The final activation capacity of DCs towards T cells is collectively destined by numerous surface regulatory molecules, which provide immunoactivating or immunosuppressive signals. The most important costimulatory molecules are structurally related glycoproteins CD80 (B7-1) and CD86 (B7-2), which augment the signal for T-cell activation (21). Membrane-bound CD83, a maturation marker for DCs in the peripheral circulation, possesses as well stimulatory capacity. Immune checkpoint molecule B7-H1 (CD274, PD-L1) is a crucial immunosuppressive glycoprotein on the DC surface and is important for maintaining self-tolerance and modulating the strength of immune response (22). However, the expression of B7-H1 could be counterproductive in the course of cancer (23), since it reduces stimulatory capacity of DCs (24, 25).

Several signaling pathways were described to modulate the expression of regulatory molecules and activating properties of DCs. The Janus family of tyrosine kinases (JAK), together with signal transducer and activator of transcription (STAT) family, constitutes a crucial signal transduction pathway that negatively regulates DC activation potential, also in malignancies (26). Particularly the activation of STAT3 is required to suppress DC function (27), and STAT3-deficient DCs demonstrate enhanced immune activity (28).

On the gene control level, microRNAs (miRNA/miR: small single-stranded, noncoding RNAs) are crucial post-transcriptional regulators of many aspects of DC biology, including development, maturation, surface molecule expression and released cytokines (29). Among discovered functional miRNAs in DCs, miR-155 has emerged as a particularly prominent player, proved to positively regulate the maturation markers and the production of several pro-inflammatory cytokines upon DC stimulation (30, 31).

In the present study, we examined the effect of sulforaphane on the expression of DC regulatory molecules and on the consequent activation capacity of DCs towards T cells in normal conditions and in context of pancreatic cancer cell-derived antigens. Next, we explored the underlying molecular mechanisms and demonstrated that JAK/STAT3 pathway and miRNA signaling could mediate the observed immunoregulatory effect of sulforaphane on DCs.

## Materials and Methods

### Tumor Lysate Preparation from Established Cell Lines

The established human pancreatic cancer cell line BxPc-3 was obtained from the American Type Culture Collection (Manassas, VA, USA), and authenticated by a commercial service (DSMZ, Braunschweig, Germany). The cells were cultured in DMEM (PAA, Pasching, Austria), supplemented with 10% FCS (Sigma-Aldrich GmbH, St. Louis, USA) and 25 mmol/L HEPES (PAA). Mycoplasma infection was excluded by performing the PlasmoTest™ (InvivoGen Europe, Toulouse, France) monthly. Tumor lysate was prepared using the standard freeze-thaw method with 4 consecutive cycles (32). After centrifugation, the supernatant was used as a source of tumor antigens.

### Peripheral Blood Mononuclear Cells and Monocyte-Derived Dendritic Cells

Fresh plasma and peripheral blood mononuclear cells (PBMCs) were obtained from human whole blood from healthy donors by Biocoll (Biochrom GmbH, Berlin, Germany) density gradient centrifugation. Monocyte-derived dendritic cells (MoDCs) were generated *in vitro* as described previously (33). Briefly, PBMCs were washed 3× with PBS (Invitrogen, USA) and re-suspended in RPMI-1640 medium (Merck KGaA, Darmstadt, Germany). PBMCs at a concentration of 1×10^6^/cm^2^ were incubated for 1–1.5 h, at 5% CO_2_ and 37°C. Culturing plates with the cells attached to the bottom were rinsed 3× with prewarmed RPMI-1640 medium to obtain adherent monocytes. Alternatively, instead of 1–1.5 h incubation, washed PBMCs were subjected to the MACS® Isolation with CD14 Microbeads for the purification of monocytes.

The adherent cells or beads-isolated CD14^+^ monocytes were incubated in RPMI-1640 medium supplemented with 1.5% autologous plasma, 400 IU/ml GM-CSF and 500 IU/ml IL-4 (both from ImmunoTools GmbH, Friesoythe, Germany) at 5% CO_2_ and 37°C (day 0). The non- and semi-adherent cells in the culturing plates were collected on day 5 or 6 as MoDCs and were used for experiments. Throughout the 5–6-day culturing and the following experiments, the cell morphology was daily analyzed with an invert light microscope (Olympus, Tokyo, Japan). The quality of obtained MoDCs was confirmed by flow cytometry to ensure the absence of CD14 expression.

### Magnetic-Activated Cell Sorting

Magnetic-activated cell sorting (MACS®) was applied for the isolation of native myeloid dendritic cells (mDCs) as well as monocytes. CD1c^+^ Dendritic Cell Isolation Kit and CD14 MACS® MicroBeads Kit (both from Miltenyi Biotech GmbH, Bergisch Gladbach, Germany) were used according to the manufacturer’s instructions.

### Reagents and DC Treatment

Sulforaphane (Sigma-Aldrich GmbH) was dissolved in DMSO to a 100 mM stock solution and stored in aliquots at -20°C. Each aliquot was used only once immediately after thawing and DMSO was applied as vehicle control. DC maturation was achieved by a standard cytokine cocktail containing TNF-α, IL-1β, IL-6, and PGE2 (all purchased from ImmunoTools GmbH). Signal transduction inhibitors targeting JAK, STAT1/3/5, p38, ERK, and PI3K were applied to chemically block the respective pathways: SB203580 (p38 MAPK inhibitor, Cell Signaling Technology, Frankfurt am Main, Germany), U0126 (MEK1/2 inhibitor, Cell Signaling Technology), LY294002 (PI3K inhibitor, Cell Signaling), CAS457081-03-7 (Calbiochem^®^ JAK inhibitor, Merck KGaA), Cucurbitacin/JSI-124 (Calbiochem^®^STAT3 inhibitor, Merck KGaA), S14-95 (STAT1 inhibitor, Abcam, Cambridge, USA), and STAT5 inhibitor Nʹ-((4-Oxo-4H-chromen-3-yl) methylene) nicotinohydrazide (Calbiochem^®^STAT5 inhibitor, Merck KGaA). The final concentrations of the solvents in medium were 0.1% or less.

### Proliferation Assay

PBMCs were labeled with Proliferation Dye eFluor™670 (Thermo Fisher, Darmstadt, Germany, referred to as FluorDye in the following text) according to the manufacturer’s instructions. Subsequently, DCs were cocultured with PBMCs in 96-well round-bottom plates at a fixed ratio, with the addition of CD3/CD28 purified antibodies (Thermo Fisher) and incubated at 37°C and 5% CO_2_ for 5 days. The cultures were harvested, stained if needed with fluorescence-coupled monoclonal antibodies (mAbs) (CD69, CD25, CD3, CD4, CD8), and examined by flow cytometry. The proliferation of T cells was quantified by the distribution of the FluorDye intensity among dividing cells.

### Western-Blot Analysis

Whole-cell protein extracts were prepared and detected by western blot analysis using 5 µg protein per sample as previously described (6). The following antibodies were used: Phospho-STAT3 (Tyr705) (D3A7) (Cell Signaling Technology), β-actin (A1978, Sigma-Aldrich GmbH). ImageJ software was used to analyze the pixel intensities of the western blot bands by Gel Analysis function.

### Flow Cytometry Analysis

Flow cytometry was performed to assess the expression of DC- and T-cell surface markers as well as FluorDye signal intensity as described previously (34). For cell surface staining, directly labeled mAbs including CD14-FITC, CD25-V450, CD69-APC-Cy7, CD83-APC, CD86-PE, B7-H1-PE, CD3-PE, CD4-APC-H7, CD8-V500 (all from BD Biosciences, Heidelberg, Germany), and CD80-FITC (Beckman Coulter Inc, Indianapolis, USA) were used. In brief, cells were collected, washed with PBS and resuspended in FACS buffer (PBS supplemented with FCS, EDTA). The cell suspension was blocked for 10 min with human FCR blocking reagent (Miltenyi Biotech GmbH), incubated for 30 min with antibodies, washed 2× and re-suspended in FACS buffer. Afterwards, each sample was analyzed by a BD FACSCanto II flow cytometer and FlowJo software (both BD Biosciences). Results were expressed as medium fluorescence intensity (MFI) or percentage of cells.

### microRNA Isolation and Profiling

The miRNeasy^®^ Mini Kit (Qiagen, Hilden, Germany) was used for the extraction of DC-derived miRNA according to the manufacturer’s instructions. Microarray profiles were obtained using a human Affymetrix GeneChip miRNA 4.0 Array (Affymetrix Technologies, Santa Clara, CA), in triplicates. Following the manufacturer’s protocol, 1 µg of total RNA was labeled with the FlashTag Biotin HSR RNA labeling kit (Affymetrix, Santa Clara, CA). The GeneChip Fluidics Station 450 was used for washing and staining (Thermo Fisher). Microarray chips were read on the GeneChip Scanner 3000 (Thermo Fisher). Data was imported and analyzed using the expression console software package from Affymetrix and the JMP Genomics Software from SAS (SAS Institute, Cary, NC, USA). The raw fluorescence intensity values were normalized by applying quantile normalization. The raw and normalized data are deposited in the Gene Expression Omnibus database with the accession number GSE155246. An One Way ANOVA was performed to identify differentially expressed miRNAs using a commercial software package SAS JMP Genomics, version 7 (SAS Institute). A false positive rate of 0.05 with FDR correction was taken as the level of significance.

The online platform MiRPathDB v2.0 (35) was used to predict the pathways regulated by particular miRNAs. Another online platform miRWalk (36) was used to predict interactions between miRNAs and genes. The experimentally validated online database mirTarBase (37) was used to confirm the detected gene targets of the identified miRNA candidates. Encori website (38) with functions microRNA Survival Analysis and Gene Survival Analysis based on TCGA data was used for the survival correlations.

### microRNA Transfection

*Mir*Vana™ mimics, inhibitors and corresponding nonsense miRNAs (mock) (Thermo Fisher) were transfected at a concentration of 30 nM into DCs using Lipofectamine 2000 (Thermo Fisher) with a reverse transfection method according to the manufacturer’s instructions. The 5´-3´ sequence of hsa-miR-155-5p is UUAAUGCUAAUCGUGAUAGGGGUU.

### Quantitative Real Time PCR

TaqMan^®^ Small RNA Assays (Thermo Fisher) was used according to the manufacturer’s instructions. The expression of miR-155-5p (Assay ID: 467534_mat, Thermo Fisher) was evaluated using the TaqMan^®^ Universal Master mix (Thermo Fisher) and normalized to the small nuclear RNU6B endogenous control. The reaction was conducted with the StepOne Real-Time PCR System (Applied Biosystems, Darmstadt, Germany).

### Statistical Analysis

All quantitative data are presented as the mean values ± SD (standard deviation). The experiments were performed by the use of DCs/PBMCs from at least three independent healthy donors. Following statistical tests were used: Student’s t-test, OneWay ANOVA test, distribution analysis, correlation and principal variance component analysis. P ≤ 0.05 was considered statistically significant, *P ≤ 0.05, **P ≤ 0.01. For the miRNA microarray data, the JMP software (SAS Institute) was used.

## Results

### Sulforaphane Limitedly Affects DC Viability

**Figure 1:**
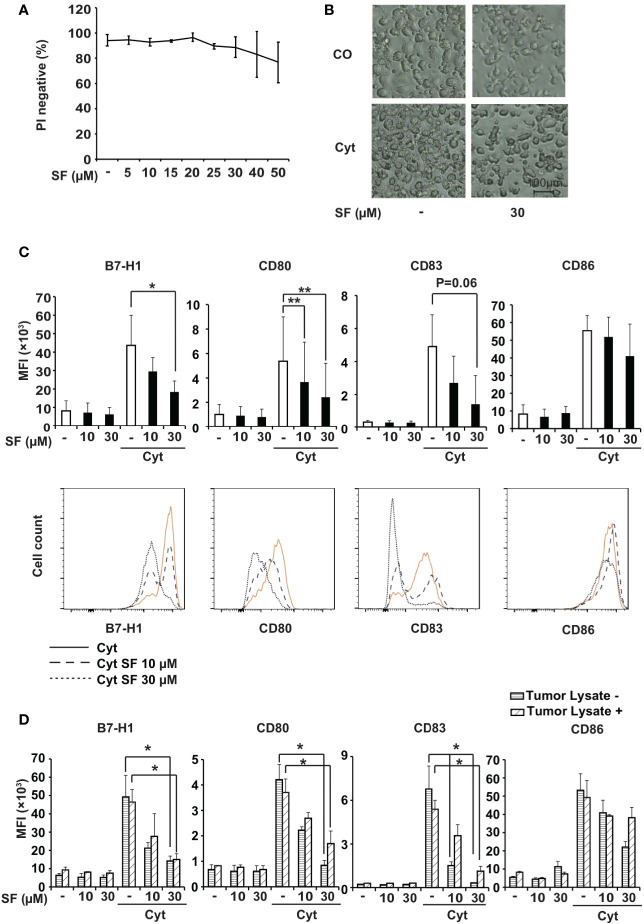
Sulforaphane limitedly affects viability and downregulates surface molecules in monocyte-derived dendritic cells (MoDCs). **(A)** MoDCs were treated with vehicle control or with different concentrations of sulforaphane (SF) as indicated, and the viability was examined 48 h later by propidium iodide (PI) staining and flow cytometry. **(B)** Immature (CO) or mature (Cyt) MoDCs were treated with 30 µM sulforaphane or vehicle control for 48 h. The morphology was documented by microscopy at 100× magnification. Representative images are shown. **(C)** MoDCs were pretreated with 10 µM, 30 µM sulforaphane or vehicle control for 30 min and treated with cytokine cocktail (Cyt) or vehicle control for 48 h. Afterwards, cells were labeled with B7-H1-PE, CD80-FITC, CD83-APC, or CD86-PE antibody and analyzed by flow cytometry and FlowJo software. The data are presented in mean fluorescence intensity (MFI) ± standard deviation (SD). The histograms below provide the data from one representative experiment, n = 4, *P ≤ 0.05, **P ≤ 0.01 **(D)** BxPc-3 cells were frozen in a dry ice bath and thawed at 37°C water bath for 4 consecutive cycles. The BxPc-3 lysate (Tumor Lysate +) or culturing medium (Tumor Lysate -) was used to treat MoDCs for 24 h. Afterwards, the cells were treated as in 1C. The data are presented in MFI ± SD, n=3, *P ≤ 0.05, **P ≤ 0.01.

To establish the sulforaphane working concentration for DCs, we performed a dose-response viability assay. MoDCs were treated with sulforaphane from 5 to 50 µM because these concentrations are in the range of observed antitumor effects (with 10 and 30 µM) in established PDA cell lines (6). After 48 h treatment, the MoDCs were stained with propidium iodide and investigated by flow cytometry. Cell viability was not significantly repressed within 30 µM, but a modest dose-dependent inhibition of cell viability was observed starting at 25 µM (**Figure 1A**). This result was confirmed in the usually more sensitive myeloid (m)DCs and demonstrated a slightly decreased viability already at a sulforaphane concentration of 5 µM, but no significant changes were observed (**Figure S1A**).

Maturation of DCs is usually accompanied by a morphological alteration, from a circular shape to possessing cytoplasmic protrusions. In our experiment, while maturation of DCs with the cytokine cocktail led to the expected morphological changes, treatment with 30 µM sulforaphane did not alter single-cell microscopic features of both immature and mature DCs, although causing a lower DC density (**Figure 1B**). Due to these results, sulforaphane was applied in all following experiments at concentrations of 10 µM and 30 µM.

### Sulforaphane Downregulates the Expression of DC Surface Molecules

To address the immunomodulatory effect of sulforaphane on DCs, we examined the influence of sulforaphane on the expression of the regulatory molecules B7-H1, CD80, CD83, and CD86. MoDCs were pretreated with sulforaphane or were left untreated followed by the addition of a cytokine cocktail, which induces maturation of DCs and the expression of regulatory molecules. As analyzed by flow cytometry, sulforaphane significantly downregulated the expression of B7-H1 and CD80 in mature DCs, and most likely also of CD83 (p=0.06). We also observed a tendency to downregulation of CD86, although this was not statistically significant (**Figure 1C**).

Likewise, sulforaphane treatment of freshly isolated blood mDCs demonstrated a significant reduction of B7-H1 and a strong trend for downregulation of CD80 and CD83 regulatory molecules, although these differences did not achieve statistical significance (**Figure S1B**).

To test whether sulforaphane could also affect the expression of DC regulatory molecules in presence of pancreatic cancer antigens, we prepared tumor antigens from the established human BxPc-3 PDA cell line and incubated them with MoDCs. These “tumor-pulsed” DCs and native DCs were then treated with sulforaphane in presence or absence of cytokine cocktail and subjected to flow cytometry. Sulforaphane significantly downregulated the expression of B7-H1, CD80, and CD83 molecules. The reduction of CD86 expression was not statistically significant (**Figure 1D**). Thus, sulforaphane modulates the expression of DC regulatory molecules in presence and absence of pancreatic cancer antigens, suggesting that sulforaphane could influence the costimulation/suppression ratio.

### Sulforaphane Improves the Stimulatory Capacity of DCs

**Figure 2:**
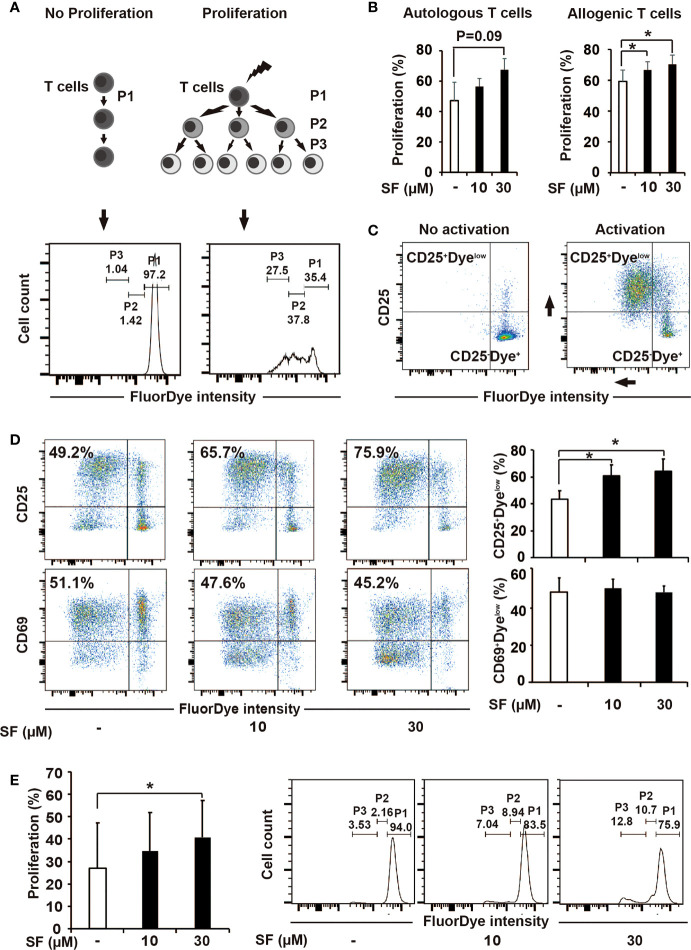
Sulforaphane improves stimulatory capacity of dendritic cells (DCs). **(A, B)** The principle and evaluation of proliferation assay. **(A)** Difference in the activation ability towards T cells was assessed by the distribution of FluorDye among proliferating cells. The representative histograms show the gating strategy of a non-proliferating (P1) and proliferating T-cell populations (P2 and P3) in negative and positive control. The numbers indicate different percentages of each portion from one representative experiment. **(B)** Mature monocyte-derived dendritic cells (MoDCs) were treated with sulforaphane (SF) at indicated concentrations or vehicle control for 48 h. The same amount of DCs were co-incubated with autologous or allogenic FluorDye-labeled peripheral blood mononuclear cells (PBMCs) at ration 1:10 for 5 days, with the addition of CD3/CD28 purified antibodies. The proliferation of T cells was analyzed by flow cytometry and FlowJo software. The sum of P2 and P3 percentage was used as a Proliferation percentage (%), n = 4. **(C, D)** The principle of assessment and evaluation of T-cell late activation marker CD25. **(C)** The dot plot on the left indicates a non-proliferating T-cell population (negative control), composing to a large extent CD25^-^Dye^high^ population. During T-cell activation (positive control), FluorDye distributes among daughter cells leading to the FluorDye signal shift to the left in X-axis, and the CD25 shift in Y-axis, forming CD25^+^Dye^low^ population. Representative dot plots are shown. **(D)** Co-incubation of DCs and PBMCs was performed as in 2B. Cells were labeled with CD25-V450 and CD69-APC-Cy7 antibodies and analyzed by flow cytometry and FlowJo software. The data are presented as mean ± SD. Dot plots provide the data from one representative experiment, n = 4. **(E)** Sulforaphane affects stimulatory capacity of DCs in presence of pancreatic cancer antigens. Tumor lysate was prepared and used to treat MoDCs for 24 h. MoDCs were matured and treated with sulforaphane at indicated concentrations or vehicle control for 48 h. The co-incubation was performed and analyzed as shown in **(B)**. Representative histograms are shown on the right, n = 4. *P ≤ 0.05.

To examine the effect of sulforaphane on the activation capacity of DCs toward T cells, we performed a proliferation assay. Human PBMCs were labeled with FluorDye, preactivated or not with CD3/CD28 antibodies followed by co-incubation with MoDCs that have been treated with sulforaphane for 48 h or were left untreated. After 5 days, cells were collected and analyzed by flow cytometry with the gating on lymphocytes. The majority of gated lymphocytes were confirmed to be CD3^+^ cells, indicating a T-cell population (**Figure S2**). Based on the FluorDye intensity, we evaluated non-proliferating (P1) and proliferating (P2 and P3) cells (**Figure 2A**). We observed that sulforaphane-treated DCs led to a sulforaphane dose-dependent, higher proliferation of T cells, compared to their non-treated counterparts (**Figure 2B**).

We additionally examined the surface expression of CD25, a marker for late T-cell activation, and found that its expression on total T cells was higher upon 5-day co-cultivation with sulforaphane-treated DCs, compared to non-treated DCs (**Figures 2C, D**). The CD69 expression was non-altered, as expected due to its role as an early T-cell activation marker that is already partially downregulated to this time point (**Figure 2D**). To get knowledge whether sulforaphane-treated DCs affect both CD4^+^ and CD8^+^ conventional T cells, we additionally stained the cocultured PBMCs with CD4 and CD8 mAbs, followed by flow cytometry analysis. Both CD4^+^ and CD8^+^ T cells in the cocultures were proliferating better in response to sulforaphane-treated DCs (**Figure S3**).

To examine whether the effect of sulforaphane-DCs on T-cell proliferation would be similar in the presence of pancreatic cancer antigens, proliferation assay was repeated with tumor-pulsed DCs, which were produced by adding tumor antigens into DC culturing medium for 24 h. Then MoDCs were matured and treated with sulforaphane at indicated concentrations or vehicle control for 48 h. As shown in **Figure 2E**, sulforaphane-treated tumor-pulsed DCs stimulated proliferation of T cells better than non-treated counterparts. These results indicate that sulforaphane can increase the stimulatory capacity of DCs in context of PDA.

### JAK/STAT3 Signaling Is Involved in the Regulatory Effect of Sulforaphane

**Figure 3:**
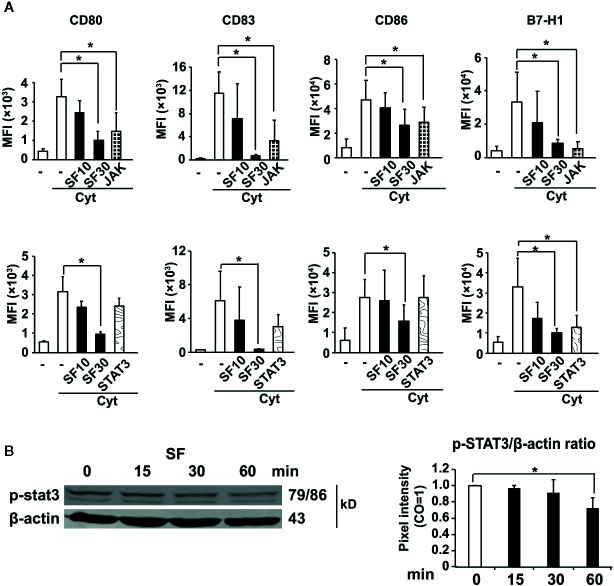
Sulforaphane-reduced phosphorylation of STAT3 leads to the downregulation of B7-H1. **(A)** Monocyte-derived dendritic cells (MoDCs) were pretreated with vehicle control, indicated concentrations of sulforaphane (SF) or respective pathway inhibitors for 30 min. Then cytokine cocktail (Cyt) or vehicle control was added into the cultures. Cells were collected 48 h later, labeled with B7-H1-PE, CD80-FITC, CD83-APC, or CD86-PE antibody and analyzed by flow cytometry and FlowJo software. Data are presented in Mean fluorescence intensity (MFI) ± SD, *P ≤ 0.05, n = 4-7. **(B)** MoDCs were treated with sulforaphane or vehicle control for 15 min, 30 min and 60 min, activated by cytokine cocktail, the proteins were harvested and phosphorylation of STAT3 was examined by western blot. The expression of β-actin served as a control for equal total protein. The band intensities (pixel intensities) were evaluated using ImageJ software after normalization to β-actin, n = 3, *P ≤ 0.05.

To explore the underlying molecular mechanisms for our observations, we used specific inhibitors for signaling pathways, which have been shown previously to control the expression of regulatory molecules and to affect the activation capacity of DCs, including JAK/STAT, p38, ERK, and PI3K signaling (39–43). Particularly we were interested in JAK/STAT3 pathway, since the improved activation capacity of DCs as well as the reduced expression of suppressive B7-H1 molecule was shown to be dependent on the inhibition of STAT3 phosphorylation. DCs were pretreated with JAK/STAT3, STAT1/5, p38, ERK42/44, PI3K inhibitors, or sulforaphane, then matured by a cytokine cocktail. After 48 h, the expression of regulatory molecules was examined by labeling DCs with fluorescent mAbs towards CD80, CD83, CD86, and B7-H1 and analyzing by flow cytometry. We found that inhibition of JAK significantly mimicked the sulforaphane-mediated downregulation of all investigated regulatory molecules, CD80, CD83, CD86, and B7-H1, and STAT3 inhibitor significantly reproduced the effect of sulforaphane on B7-H1 (**Figure 3A**). Other significant correlations between pathway inhibition and reduced expression of regulatory molecules were observed between PI3K and CD80/CD83/CD86, p38 and CD83, STAT1 and CD83, as well as ERK and CD83/B7-H1 (**Figure S4**). STAT5 inhibition did not show any correlation. To evaluate whether sulforaphane might directly inhibit JAK/STAT3 signaling, we treated cytokine-activated DCs with sulforaphane for 15 to 60 min and examined the phosphorylation of STAT3 by western blot analysis and evaluation of the pixel intensity of bands by ImageJ software. Sulforaphane could moderately inhibit STAT3 phosphorylation starting at 30 min after the treatment, and acting even more pronounced and significantly at 60 min (**Figure 3B**). Thus, sulforaphane-mediated downregulation of STAT3 phosphorylation may be involved in inhibition of the immune-suppressive B7-H1 molecule, whose expression correlates with the level of STAT3 phosphorylation (39).

### miR-155-5p and miR-194-5p Could Be Accountable for the Effect of Sulforaphane on DCs

**Figure 4:**
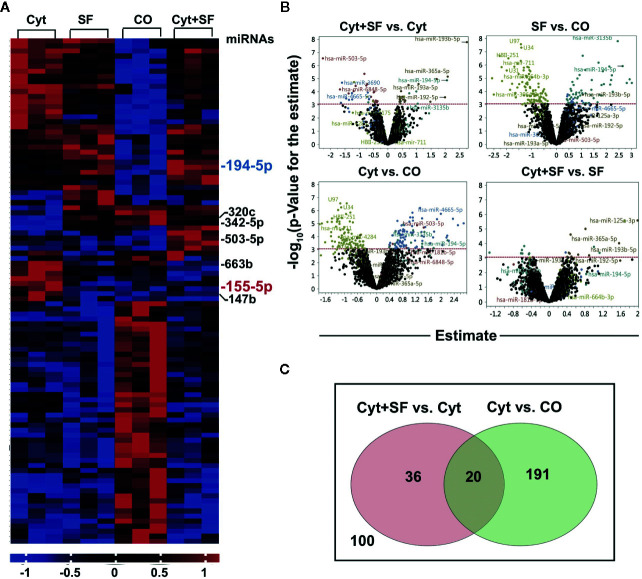
Sulforaphane differentially regulates microRNAs (miRNAs) in dendritic cells (DCs). **(A)** Monocytes were isolated from peripheral blood mononuclear cells (PBMCs) using CD14 MicroBeads and monocyte-derived dendritic cells (MoDCs) were produced *in vitro*. MoDC were treated with 30 µM sulforaphane (SF) or vehicle control for 30 min, then cytokine cocktail (Cyt) or vehicle control were added into the cultures for 48 h followed by miRNA extraction. The miRNA array analysis and bioinformatic evaluation were performed to select the most significantly regulated miRNAs. The heat map presents most significantly differently expressed miRNAs across 4 groups. The red colors indicate high expression, and the blue colors indicate low expression within a scale from 1 to -1. **(B)** The four Volcano plots demonstrate the distribution of differentially regulated miRNAs in respective comparisons. On the y-axis, the –log10 p-Value (the grade of significance) is plotted; on the x-axis, the estimate for the comparison (fold change) is represented. **(C)** Venn diagram shows differently regulated miRNAs candidates between the groups (Cyt+SF) and (Cyt) as well as between the groups (Cyt) and (CO). The overlapping 20 miRNAs are the promising candidates for the observed sulforaphane effect on DCs.

To further elucidate the underlying mechanism of our findings, we focused on the involvement of sulforaphane-induced miRNA signaling (44). MACS® CD14 MicroBeads Kit was used to isolate high purity monocytes from PBMCs to generate MoDCs. These high-quality MoDCs were pretreated with sulforaphane or vehicle control for 30 min, followed by maturation with a cytokine cocktail or were left immature. The miRNA from the resulting four groups, namely control, sulforaphane-treated, cytokine-treated, and cytokine- and sulforaphane-treated, was harvested and subjected to miRNA array and bioinformatic analysis. We identified the top 100 most significantly up- and downregulated miRNAs (**Figure 4A**). The distribution of differentially regulated miRNAs between the groups is demonstrated by volcano plots, where the x-axis shows the fold change of regulated miRNAs, and the y-axis the grade of significance (**Figure 4B**).

The most significant differences in the expression of regulatory molecules and DC activation capacity were observed between the control and cytokine-treated MoDCs and between the cytokine-treated and cytokine- and sulforaphane-treated cells. Therefore, we created a Venn diagram, representing 211 miRNA candidates who changed significantly between the control and cytokine-treated MoDCs and 56 candidates who changed significantly between the cytokine-treated and cytokine- and sulforaphane-treated cells. The two circles of candidates were matched, resulting in 20 miRNAs predicted to be regulated by both cytokines and sulforaphane (**Figure 4C**, **Supplementary Table 1**). Most importantly, these 20 miRNAs were all among the top 30 candidates of the heat map.

**Figure 5:**
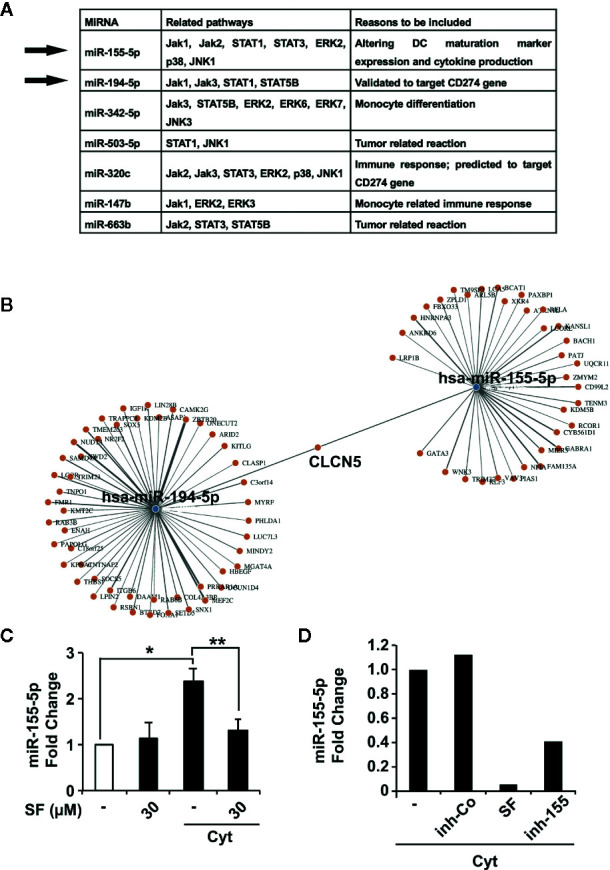
miR-155-5p and miR-194-5p are accountable for the effect of sulforaphane on dendritic cells (DCs). **(A)** The prediction of miRNA targets using online resources (http://www.mirdb.org, http://mirtarbase.mbc.nctu.edu.tw) together with published data resulted in the identification of seven most promising candidates and selection of miR-155-5p and miR-194-5p. **(B)** Identification of joint and unshared target genes of miR-155-5p and miR-194-5p miRNAs. Target Mining option of the mirWalk online platform was used for miR-155-5p and miR-194-5p. Predicted probability was set to 0.9, TargetScan and MirDB were included. **(C)** Immature or mature (Cyt) monocyte-derived dendritic cells (MoDCs) were treated with vehicle control or 30 µM sulforaphane (SF) for 48 h, followed by RNA isolation, and RT-qPCR was used to assess the content of miR-155-5p in different RNA samples, n = 3 **(D)** MoDCs were pretreated with vehicle control (-) or 30 µM sulforaphane, lipotransfected with inhibition control (inh-Co) or miR-155-5p inhibitor (inh-155), then activated with cytokine cocktail (Cyt) for 48 h, followed by RNA isolation. RT-qPCR was used to assess the content of miR-155-5p in different RNA samples. *P ≤ 0.05, **P ≤ 0.01.

To further reduce these 20 candidates, we performed a literature search and an *in silico* analysis using the online databases MiRPathDB v2.0 and miRTarBase (http://www.mirdb.org, http://mirtarbase.mbc.nctu.edu.tw), as well as the online platform mirWalk (http://mirwalk.umm.uni-heidelberg.de). The selection criteria were important roles in immunity, antitumor response and control of DC regulatory molecule expression. This resulted in the identification of seven top candidates (**Figure 5A**), most of which are tightly connected to JAK/STAT pathway. Among the two most promising candidates was miR-155-5p, known to alter DC maturation marker expression and cytokine production (45), which relates to the observed sulforaphane-mediated downregulation of the costimulatory molecules CD80, CD83, and CD86. The expression of miR-155-5p was downregulated by sulforaphane (1.124928389 folds, P=0.007860369). The second top candidate miR-194-5p, which was upregulated by sulforaphane (2.060136861 folds, P= 0.004370195), was shown to target the *CD274*, the gene of immune suppressive B7-H1 protein (**Supplementary Table 1**).

Since our results predict different roles for miR-155-5p and miR-194-5p in controlling of DC regulatory molecules, we used the online platform mirWalk (http://mirwalk.umm.uni-heidelberg.de) to evaluate the target genes for these miRNAs. On mirWalk, the Target Mining option was used, TargetScan and MirDB databases were included, and the predicted probability was set to 0.9. As a result, 80 target genes were identified for miR-155-5p, 143 target genes for miR-194-5p, and only CLCN5, encoding the Cl^-^/H^+^ exchanger ClC-5 mainly expressed in the kidney, was predicted to be targeted by both (**Figure 5B**), confirming that miR-155-5p and miR-194-5p have indeed quite different spectra of targets.

To confirm the supposed sulforaphane-mediated regulation of miR-155-5p in DCs, we examined the expression levels of miR-155-5p before and after maturation by a cytokine cocktail in sulforaphane-treated and -untreated MoDCs, by qRT-PCR. The cytokine-induced expression of miR-155-5p in DCs was significantly downregulated upon sulforaphane treatment (**Figure 5C**). In the next experiment, DCs were transfected with miR-155-5p inhibitor or correspondent inhibitor control, treated with sulforaphane or left untreated, and matured by a cytokine cocktail. After 48 h, the levels of miR-155-5p expression were examined by qRT-PCR. As shown in **Figure 5D**, sulforaphane could diminish the endogenous miR-155-5p level even stronger than the transfection with the miR-155-5p inhibitor. These findings may be seen as a strong hint for the direct connection between the sulforaphane-mediated inhibition of miR-155-5p expression and the downregulation of DC costimulatory molecules. However, we were not able to directly demonstrate this relation, because of the well-known technical hindrance-unspecific activation of DCs by the transfection procedure, even with only scrambled control (data not shown).

### Sulforaphane-Mediated Modulation of miR-155-5p, miR-194-5p, and STAT3 Might Positively Influence PDA Patient Survival

**Figure 6:**
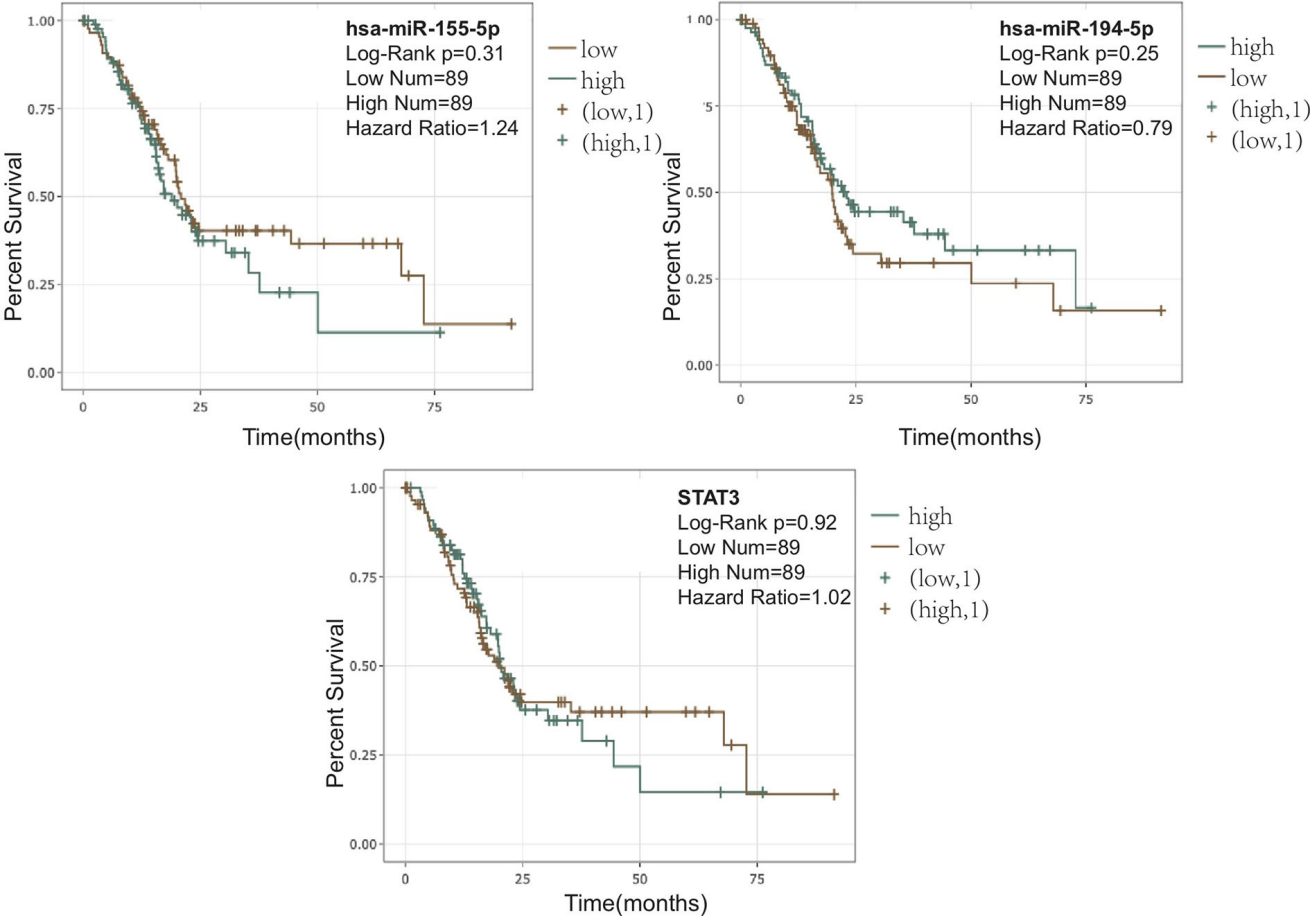
Correlation between pancreatic ductal adenocarcinoma (PDA) patient overall survival and the expression of miR-155-5p, miR-194-5p, or STAT3. Correlations and figures were generated on Encori website (http://starbase.sysu.edu.cn/) using microRNA (miRNA) Survival Analysis and Gene Survival Analysis.

The observed sulforaphane-mediated changes in pathway- and miRNA- signaling could potentially influence various oncological parameters including patient survival, when applied clinically. Therefore, we used the online platform Encori (http://starbase.sysu.edu.cn/) to investigate the relationship between PDA patient survival and the expression of miR-155-5p, miR-194-5p, or STAT3 in the whole tumor mass samples. As demonstrated in **Figure 6**, a higher content of miR-194-5p in the tumor suggests an improved outcome, while a lower expression of miR-155-5p in the tumor would ameliorate the survival. Besides, a lower STAT3 expression, related to the downregulation of STAT3 phosphorylation, could also lead to improved patient survival.

**Figure 7:**
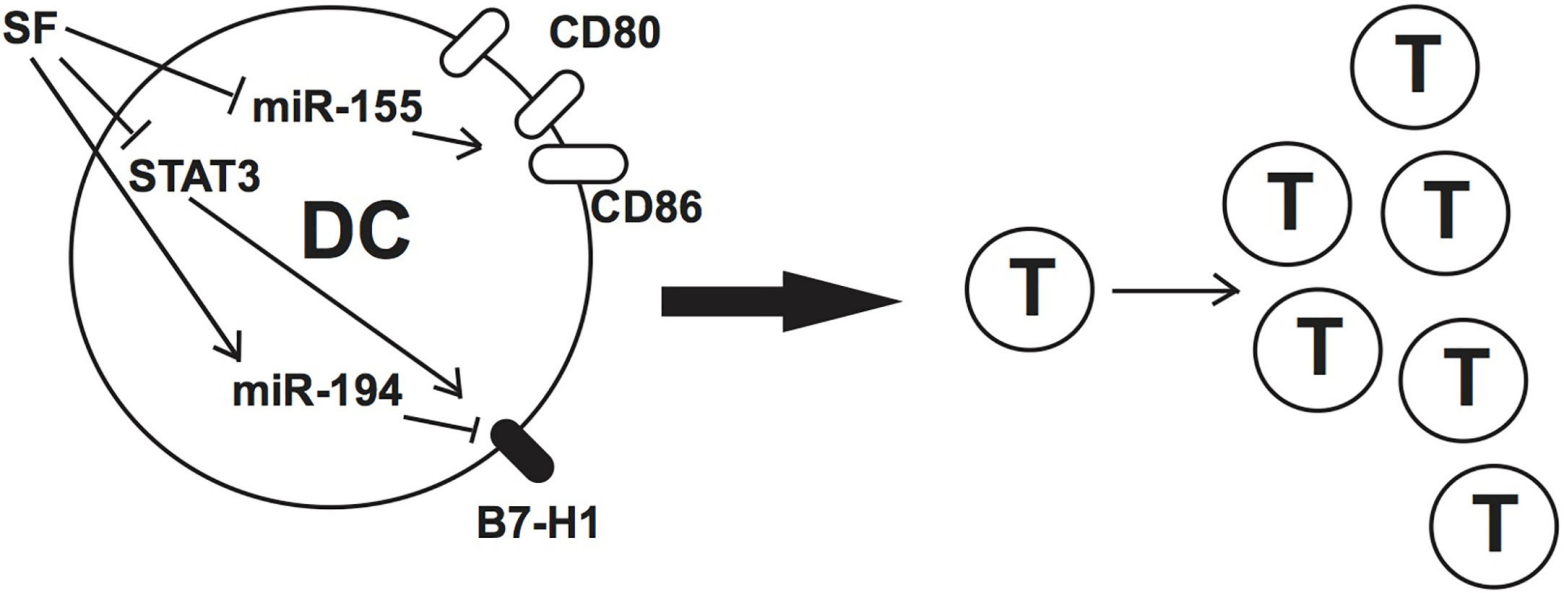
Schematic representation of our results and hypothesis.

In conclusion, sulforaphane modulates the expression of regulatory molecules and improves the activation capacity of human DCs through the downregulation of STAT3 phosphorylation leading to diminished B7-H1 expression (**Figure 7**). We showed as well a differential regulation of miRNAs by sulforaphane and identified miR-155-5p and miR-194-5p as additionally accountable for the effect of sulforaphane on DCs.

## Discussion

In this study, we demonstrated for the first time that sulforaphane improves the activation function of human DCs, which was guided by the altered expression of regulatory molecules and miRNAs, and the downregulation of STAT3 phosphorylation.

DC surface molecules CD80, CD83, and CD86 are often used as hallmarks of DC maturation and function. However, the final stimulatory capacity of DCs comprises the contributions of both costimulatory and suppressive pathways (46), whereby B7-H1 provides one of the most decisive suppressive signals (24). A previous study of Qu et al. demonstrated the effect of sulforaphane on LPS-stimulated CD80 and CD86 expression in a porcine MoDC model (47). The expression of suppressive B7-H1 molecule and the consequent stimulatory capacity of sulforaphane-treated porcine DCs towards T cells were not investigated by the authors. However, they showed that pretreatment with sulforaphane could enhance the post-activation phagocytosis of DCs, implying a stronger immune response. We demonstrated that on human DCs, sulforaphane downregulates the expression of B7-H1 suppressive proteins as well as of costimulatory molecules. For mDCs, some differences for costimulatory molecules failed to achieve significance mainly due to the pronounced heterogeneity in the activation stage of each mDC donor, which is a well-known obstacle for the work with freshly isolated primary cells. The modulation of DC regulatory molecules by sulforaphane was not accompanied by significantly affected DC viability or microscopic morphology changes. The immuno-activating potential of human DCs was not compromised but improved as is shown by proliferation and CD25 expression of T cells. We suppose that, despite both costimulatory and suppressive molecules are downregulated by sulforaphane, the strongly reduced expression of suppressive B7-H1 protein makes a more crucial input to the final stimulatory capacity of DCs than the reduction of CD80/83/86 expression, and can improve costimulation/suppression ratio. In support, a similar mode of action was described by Li et al. for other human myeloid cells, where the blockage or silencing of both the suppressive B7-H1 and the costimulatory CD80 could still significantly reduce immunosuppression of myeloid-derived suppressor cells (MDSCs) (48).

The detected ability of sulforaphane to directly reduce B7-H1 expression is quite encouraging since B7-H1 leads to the immunosuppression being present on different immune cells, such as DCs (24, 25), MDSCs (49, 50), T cells (51), and macrophages (52). Our observation let us speculate that other immune cells expressing B7-H1 could also be modulated by sulforaphane in their phenotypes and functions, and that the use of sulforaphane in cancer patients could lead to a decrease in general immunosuppression and to an improvement of antitumor response. A recent observation on human MDSCs in a glioma *in vitro* model concurs with our assumption (13). There, sulforaphane reduced MDSC frequency and B7-H1 expression in monocytes exposed to glioma-conditioned medium, consequently reducing immunosuppression and increasing T-cell proliferation.

On molecular level, we showed for the first time that sulforaphane downregulates STAT3 phosphorylation in DCs, which is consistent with their decreased expression of B7-H1 and enhanced T cell activating property. This is in agreement with Barton *et al*. who stated the stimulatory ability of DCs to be reversely correlated with the degree of STAT3 activation (53). In line, STAT3 is constitutively activated in tumor-infiltrating immune cells including DCs, and ablating STAT3 triggers the immune cells to inhibit tumor growth and metastasis (53, 54). Interestingly, the suppression of STAT3 activation by sulforaphane seems to be not cell type restricted, and, supporting our results, was recently reported in vascular endothelial cells (55), liver (56), nasopharyngeal (57), and prostate cancer cells (58).

According to the results of our pathway inhibitor assay, the repression of JAK affects the expression of CD80, CD83, CD86, and B7-H1. In contrast, the inhibition of STAT3 phosphorylation suppresses the expression of B7-H1 but not of CD80/83/86. This specific connection between STAT3 activity and B7-H1 expression was also observed in other experimental models. We showed previously that IFN-α-induced B7-H1 expression on MoDCs requires STAT3 phosphorylation (59). In mice, STAT3-dependent increase of B7-H1 expression was reported in IL-27-treated plasmacytoid DCs (60). The B7-H1 expression induced by TLR (toll-like receptor)-antagonists was modulated in immature human DCs through STAT3-dependent pathway, and in support of our findings, blocking of STAT3 activation prevented B7-H1 expression (39). In our study we did not investigate additionally the spectrum cytokines produced by DCs after sulforaphane treatment, however, our previous observations demonstrated the increase of IL-6 after upregulation of STAT3 phosphorylation. Thus, we could assume that the downregulation of STAT3 phosphorylation by sulforaphane might lead to a decrease in IL-6 production (59).

However, the downregulation of STAT3 alone is probably not exclusively responsible for the whole phenotype of sulforaphane-treated DCs. Previous investigations demonstrated that miRNAs contribute to the effect of sulforaphane on PDA (61, 62), and are also involved in the modulation of DC phenotype and function (45). Our study is the first to show that sulforaphane induces alterations in miRNA profile in DCs. We identified miR-155-5p as possibly responsible for the sulforaphane-modulated expression of costimulatory molecules on DCs. In line with our results, miR-155-deficient murine DCs showed decreased CD80/86 expression after LPS-activation (30), confirming the connection between miR-155 and DC costimulatory molecules. The downregulation of miR-155 by sulforaphane is, however, not exclusive for immune cells since it was newly observed in colon epithelial and murine microglial cells (63, 64). Our miRNA array and bioinformatics analysis also determined the sulforaphane-upregulated miR-194-5p as a top candidate for the control of B7-H1 expression. Accordingly, miR-194-5p was proved to target B7-H1 in the experimentally validated database (37) and to be upregulated by another isothiocyanate (PEITC) in prostate cancer cells (65). Thus, besides the downregulation of STAT3 phosphorylation, the upregulation of miR-194-5p by sulforaphane could be another factor responsible for the reduced B7-H1 expression. The detailed investigation of sulforaphane-induced miR-194-5p signaling in DCs and its interplay with STATs pathway are a subject of our further investigations.

The observed increased activation ability of sulforaphane-treated DCs is of high clinical importance considering the possible use of sulforaphane in combined anti-cancer therapies. Previous studies have documented the reduced activation capacity of DCs in several tumor entities including PDA (66–70) and have confirmed that enhanced T cell mediated immune response in PDA improves patient survival (71). Our results indicate the possible role of sulforaphane in improving DC activating phenotype and consequent T cell-mediated immunity, suggesting an advantageous effect of sulforaphane on adaptive immune response in addition to its previously documented antitumor activities.

The use of sulforaphane in cancer patients is still under discussion (19), mainly due to the finding that sulforaphane could directly interfere with T-cell activation (18). Despite that the overall effect of sulforaphane on cancer immunity needs further investigation, our results provide new evidence that sulforaphane does not compromise T-cell activity through DCs.

Moreover, the alteration of identified sulforaphane targets, including STAT3, miR-155-5p, and miR-194-5p, all point to a better PDA patient survival in the *in silico* analysis. Although the patient survival correlation data are based on the expression of the targets in the whole tumor mass, it is known that miRNAs can be transmitted from cell to cell in the tumor microenvironment, influencing patient outcome (72) and that tumor-infiltrating DCs are also an inherent part of tumor milieu. Thus, the modulation of these molecular targets by the use of sulforaphane may not have any negative but rather positive effect on patient outcome.

Nevertheless, the effects of sulforaphane on the immune system require further examination due to its double-edged activities. Sulforaphane might interfere differently with the various immunotherapeutic applications and a combining of sulforophane with particular cancer therapies/immunotherapies should be carefully estimated, depending on the art of therapeutic approach.

In conclusion, our data demonstrate for the first time a correlation between sulforaphane, STAT3 phosphorylation, miRNAs, regulatory molecules and the activation ability of DCs. They improve the understanding of the immunoregulatory properties of sulforaphane and justify further explorations of nutritional strategies in the co-treatment of PDA.

## Data Availability Statement

The datasets presented in this study can be found in online repositories and in **Supplementary Material**. The names of the repository/repositories and accession number(s) can be found in the article.

## Author Contributions

SK and YW: Concept and design. SK and YW: Development of methodology. YW, EP, CS, and SK: Acquisition of data. YW, CS, SK, WG, and NG: Analysis and interpretation of data. YW, IH, and SK: Writing, review, and/or revision of the manuscript. All authors contributed to the article and approved the submitted version.

## Funding

This study was supported by grants from the German Cancer Aid (Deutsche Krebshilfe 111299), German Research Council (DFG HE 3186/15-1), Heidelberger Stiftung Chirurgie, Dietmar Hopp Stiftung, Klaus Tschira Stiftung and the Hanns A. Pielenz Stiftung. Y. Wang was supported by a stipend from the China Scholarship Council.

## Conflict of Interest

The authors declare that the research was conducted in the absence of any commercial or financial relationships that could be construed as a potential conflict of interest.

## Abbreviations

DCs, dendritic cells; IFN, interferon; IL, interleukin; LPS, lipopolysaccharide; mAbs, monoclonal antibodies; MACS, magnetic activated cell sorting; mDCs, myeloid dendritic cells; MDSCs, myeloid-derived suppressor cells; miRNA/miR, microRNA; MoDCs, monocytes-derived DCs; PDA, pancreatic ductal adenocarcinoma.
