# Corrigendum: Sulforaphane promotes dendritic cell stimulatory capacity through modulation of regulatory molecules, JAK/STAT3- and microRNA-signaling

**DOI:** 10.3389/fimmu.2022.975653

**Published:** 2022-12-07

**Authors:** Yangyi Wang, Emilia Petrikova, Wolfgang Gross, Carsten Sticht, Norbert Gretz, Ingrid Herr, Svetlana Karakhanova

**Affiliations:** ^1^ Section Surgical Research, Molecular OncoSurgery Group, Department of General, Visceral and Transplantation Surgery, University of Heidelberg, Heidelberg, Germany; ^2^ Medical Research Center, Medical Faculty Mannheim, University of Heidelberg, Mannheim, Germany

**Keywords:** sulforaphane, dendritic cells, T cells, regulatory molecules, STAT3, miRNAs, pancreatic cancer

In the published article, there was an error in **Figure 2** as published. The representative images of flow cytometry in **Figure 2D**, “CD25/CD69/FluorDye _ SF 10” and “CD25/CD69/FluorDye _ SF 30” were accidently mixed up and three words “fluorescent intensity (MFI)” were misplaced in **Figure 2D** legend. The corrected **Figure 2** and its legend appear below.

**Figure 2:**
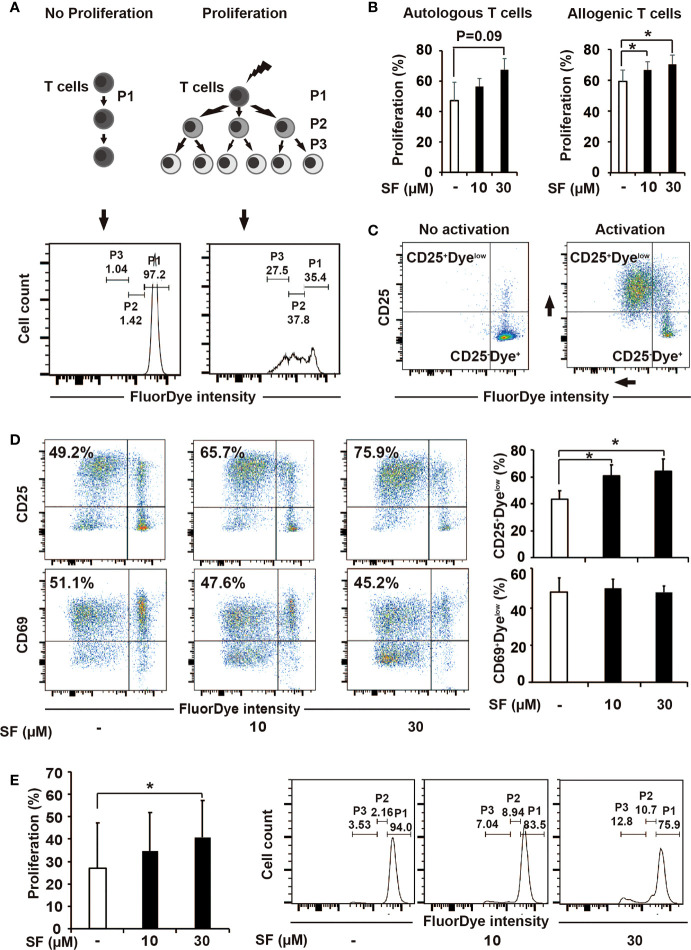
Sulforaphane improves stimulatory capacity of dendritic cells (DCs). **(A, B)** The principle and evaluation of proliferation assay. **(A)** Difference in the activation ability towards T cells was assessed by the distribution of FluorDye among proliferating cells. The representative histograms show the gating strategy of a non-proliferating (P1) and proliferating T-cell populations (P2 and P3) in negative and positive control. The numbers indicate different percentages of each portion from one representative experiment. **(B)** Mature monocyte-derived dendritic cells (MoDCs) were treated with sulforaphane (SF) at indicated concentrations or vehicle control for 48 h. The same amount of DCs were co-incubated with autologous or allogenic FluorDye-labeled peripheral blood mononuclear cells (PBMCs) at ration 1:10 for 5 days, with the addition of CD3/CD28 purified antibodies. The proliferation of T cells was analyzed by flow cytometry and FlowJo software. The sum of P2 and P3 percentage was used as a Proliferation percentage (%), n = 4. **(C, D)** The principle of assessment and evaluation of T-cell late activation marker CD25. **(C)** The dot plot on the left indicates a non-proliferating T-cell population (negative control), composing to a large extent CD25^-^Dye^high^ population. During T-cell activation (positive control), FluorDye distributes among daughter cells leading to the FluorDye signal shift to the left in X-axis, and the CD25 shift in Y-axis, forming CD25^+^Dye^low^ population. Representative dot plots are shown. **(D)** Co-incubation of DCs and PBMCs was performed as in 2B. Cells were labeled with CD25-V450 and CD69-APC-Cy7 antibodies and analyzed by flow cytometry and FlowJo software. The data are presented as mean ± SD. Dot plots provide the data from one representative experiment, n = 4. **(E)** Sulforaphane affects stimulatory capacity of DCs in presence of pancreatic cancer antigens. Tumor lysate was prepared and used to treat MoDCs for 24 h. MoDCs were matured and treated with sulforaphane at indicated concentrations or vehicle control for 48 h. The co-incubation was performed and analyzed as shown in **(B)**. Representative histograms are shown on the right, n = 4. *P ≤ 0.05.

The authors apologize for this error and state that this does not change the scientific conclusions of the article in any way. The original article has been updated.

## Publisher’s note

All claims expressed in this article are solely those of the authors and do not necessarily represent those of their affiliated organizations, or those of the publisher, the editors and the reviewers. Any product that may be evaluated in this article, or claim that may be made by its manufacturer, is not guaranteed or endorsed by the publisher.

