# Supplementary figures and images for: Sulforaphane Promotes Dendritic Cell Stimulatory Capacity Through Modulation of Regulatory Molecules, JAK/STAT3- and MicroRNA-Signaling
Source: Front Immunol. 2020 Oct 30;11:589818. doi: 10.3389/fimmu.2020.589818 (PMC7661638; doi:10.3389/fimmu.2020.589818)

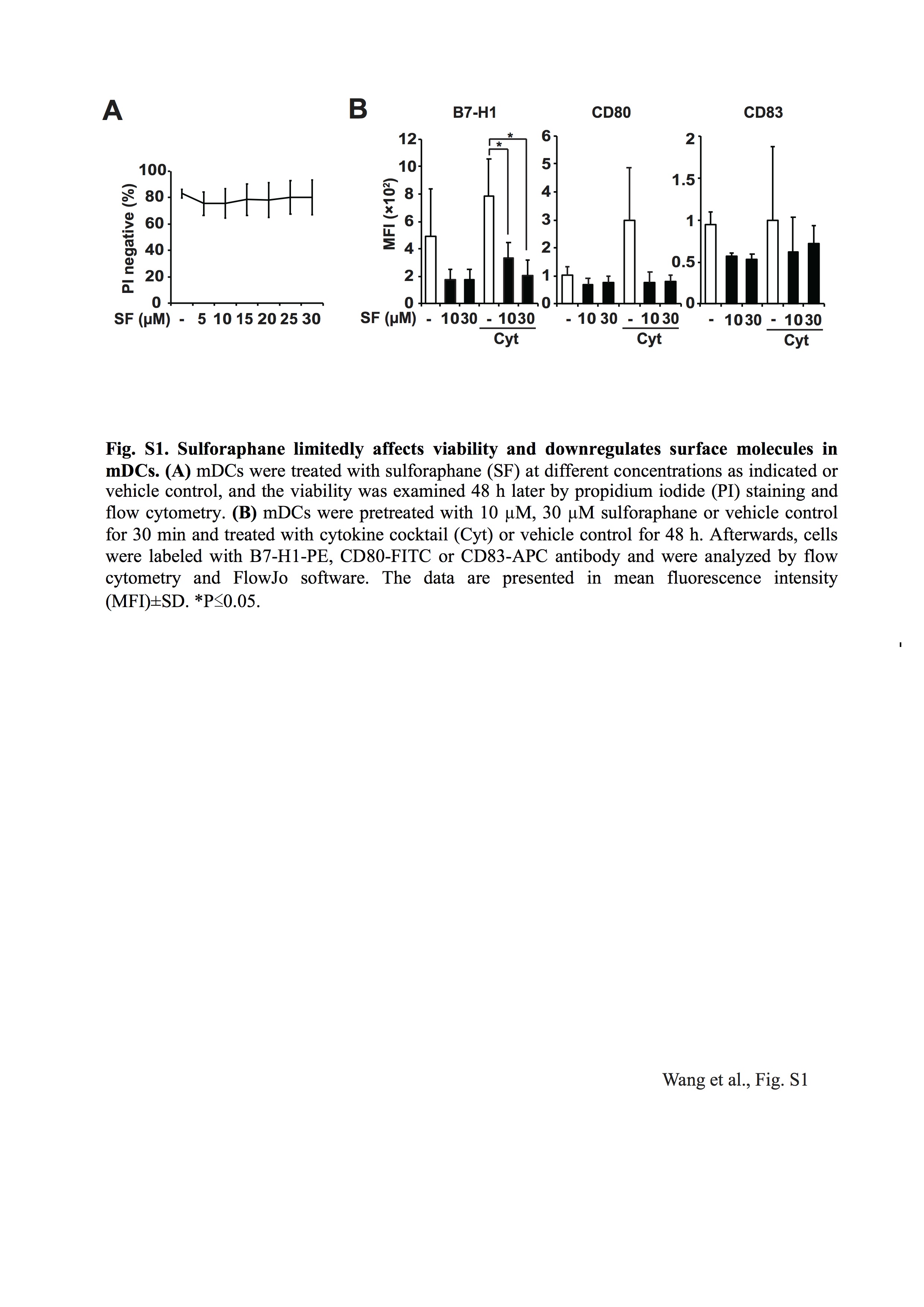

Supplement: Supplementary file 1 [file Image_1.jpeg]

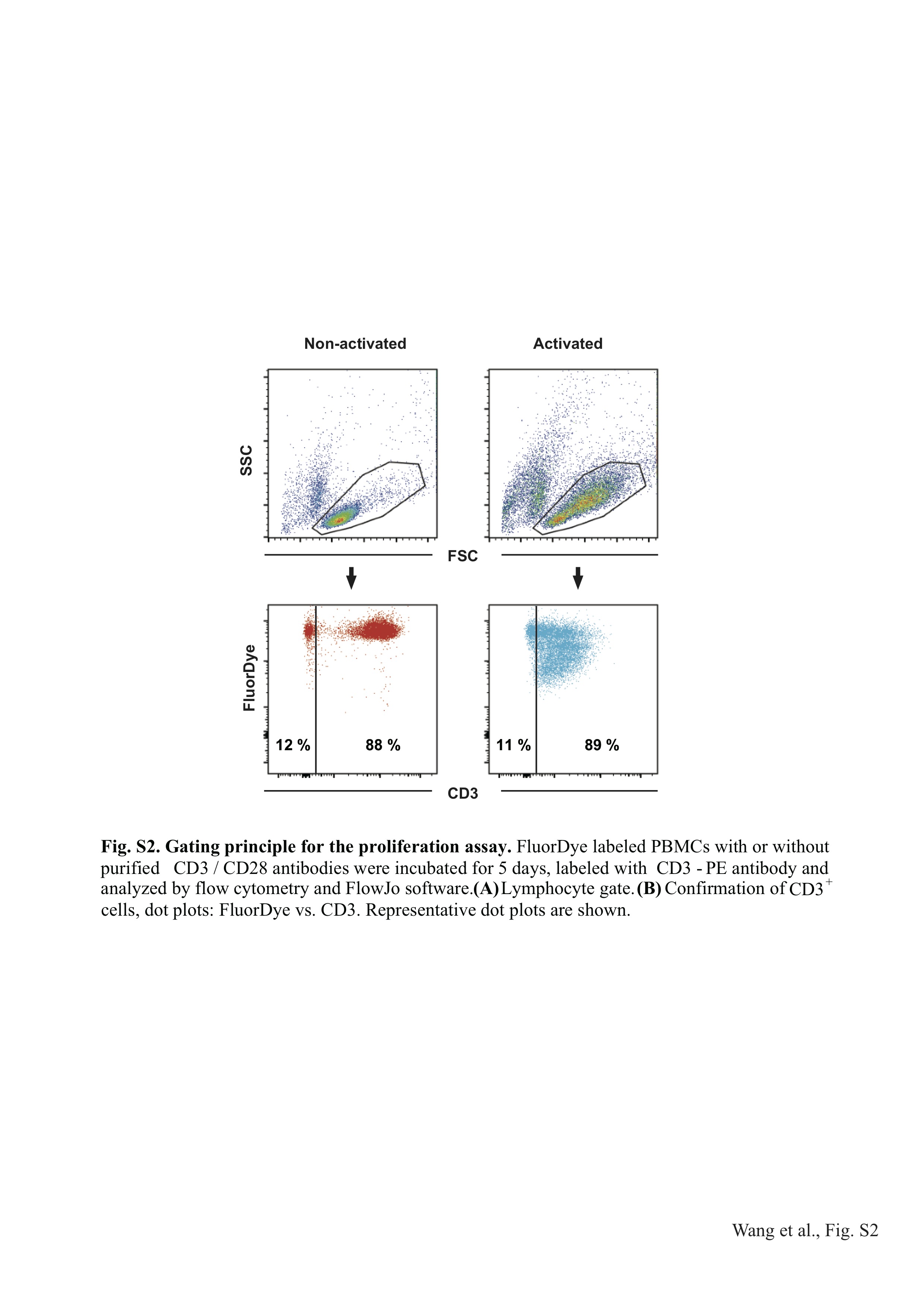

Supplement: Supplementary file 2 [file Image_2.jpeg]

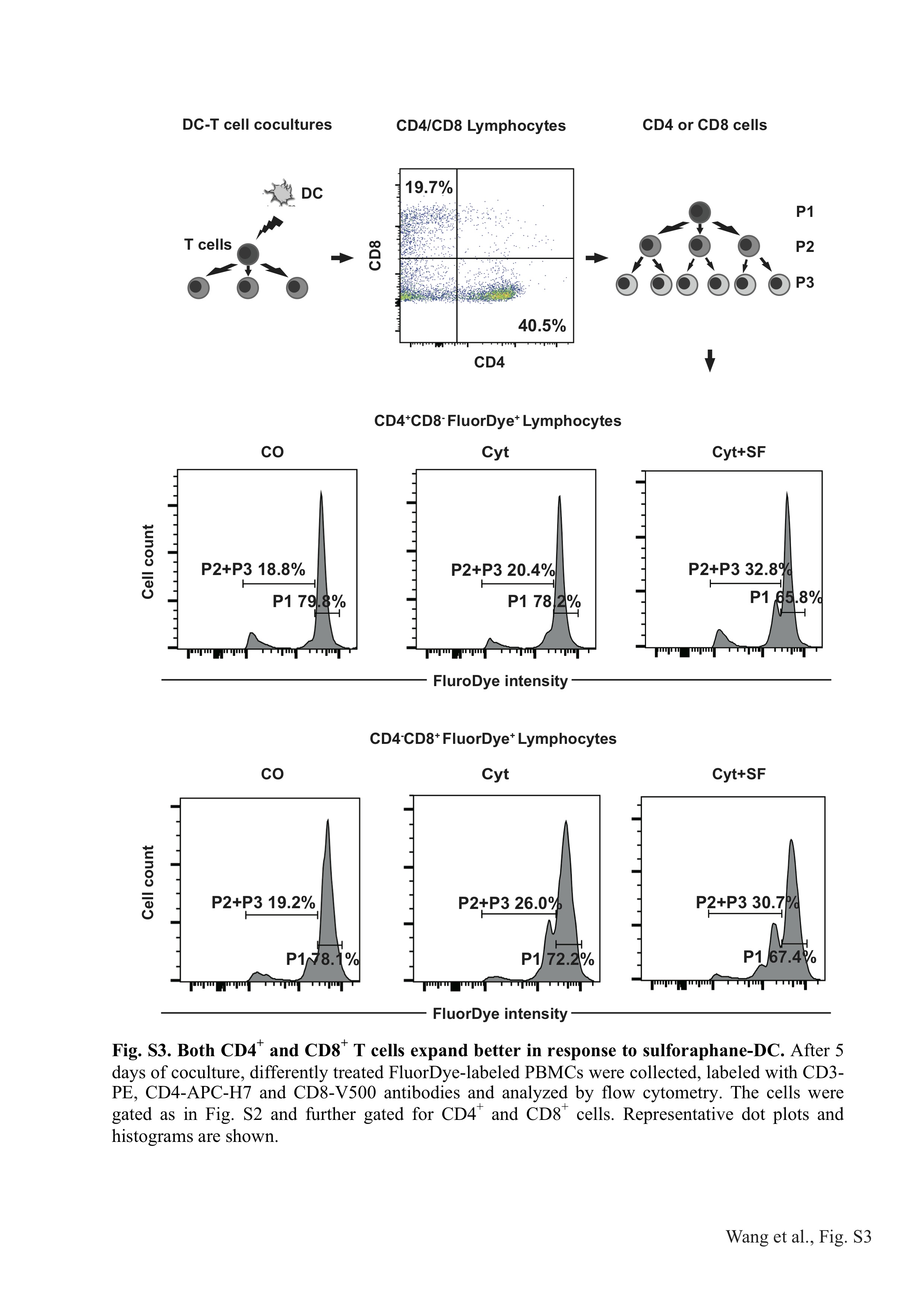

Supplement: Supplementary file 3 [file Image_3.jpeg]

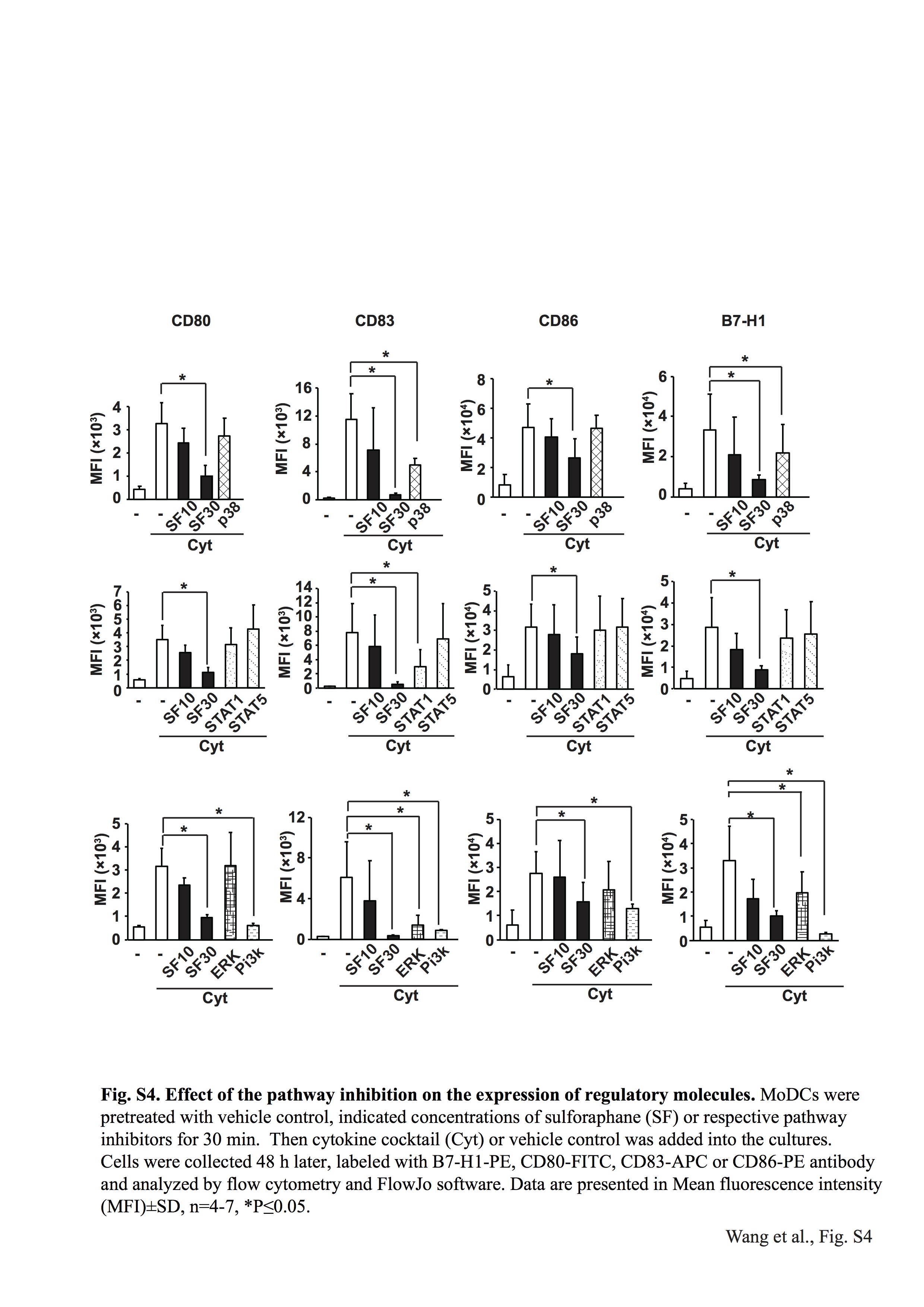

Supplement: Supplementary file 4 [file Image_4.jpeg]
