# Supplementary material for: Sulforaphane Promotes Dendritic Cell Stimulatory Capacity Through Modulation of Regulatory Molecules, JAK/STAT3- and MicroRNA-Signaling
Source: Front Immunol. 2020 Oct 30;11:589818. doi: 10.3389/fimmu.2020.589818 (PMC7661638; doi:10.3389/fimmu.2020.589818)
Supplement: Supplementary file 5 [file Table_1.pdf]

Table S1. Top 20 differentially regulated miRNAs

Transcript ID	Estimate of		Adjusted p-Value for	
	Cyt+SF vs Cyt	Cyt vs CO	Cyt+SF vs Cyt	Cyt vs CO
hsa-let-7e-5p	1.039440889	1.482498083	0.047689632	0.008843381
hsa-miR-194-5p	2.060136861	1.41447475	0.004370195	0.030061159
hsa-miR-155-5p	-1.124928389	1.251746861	0.007860369	0.004370195
hsa-miR-342-5p	0.439809639	-0.931937333	0.026795745	0.000844644
hsa-miR-503-5p	-2.266010306	1.460404972	0.000844644	0.005705924
hsa-miR-575	-1.638296869	2.701337322	0.044257575	0.003856361
hsa-miR-320c	0.422435556	-0.512628056	0.031662498	0.012785798
hsa-miR-147b	-1.245771789	1.465236692	0.031662498	0.014712477
hsa-miR-663b	-0.700901608	0.823379178	0.016021259	0.00684373
hsa-miR-3195	-1.357937028	1.782842139	0.033176794	0.008648044
hsa-miR-3690	-1.599506222	1.873587547	0.005705924	0.00267052
hsa-miR-642b-3p	-1.455504742	2.368158669	0.028395155	0.002552459
hsa-miR-4665-5p	-1.253129778	1.992361194	0.017017912	0.0017485
hsa-miR-5100	-0.81989075	0.496942778	0.002552459	0.030974972
hsa-miR-6722-3p	-0.659796889	1.121114944	0.031477295	0.002411839
hsa-miR-6768-5p	-1.194973253	0.923856183	0.013762273	0.044929516
hsa-miR-7641	-0.729697972	-0.879780833	0.023547017	0.009267814
hsa-mir-423	0.531161083	-0.606523528	0.032168343	0.017317743
hsa-mir-4800	0.562836483	-0.835954636	0.023244832	0.003279104
hsa-mir-8063	-0.494319011	0.409003556	0.015508722	0.03687645
